# Beyond Resilience: A Mixed-Method, Longitudinal Analysis of Difficulties and Positive Experiences in Older Adults During the COVID-19 Pandemic

**DOI:** 10.3390/bs16071117

**Published:** 2026-07-03

**Authors:** Carolyn M. Aldwin, Maria Kurth, Heidi Igarashi

**Affiliations:** 1 School of Human Development and Family Sciences, Oregon State University, Corvallis, OR 97331, USA; heidiigarashi@gmail.com; 2 Clearinghouse for Military Family Readiness, The Pennsylvania State University, State College, PA 16801, USA

**Keywords:** trajectories, stressors, adaptation, aging, longitudinal thematic analysis

## Abstract

Despite heightened physical risks during the COVID-19 pandemic, older adults often reported better mental health than younger adults, suggesting significant resilience. We used longitudinal qualitative data to examine how difficulties and positive experiences contributed to this resilience. Weekly COVID-related difficulties and positive experiences were collected using internet surveys over eight weeks from 247 respondents aged 51–95 (M = 71.1, SD = 7.3). Nearly all identified at least one difficulty, and 76% had problems three or more times. Longitudinal thematic analysis (LTA) revealed that most were consistent in how they described they difficulties, including problems with everyday protective activities, psychological distress, social isolation, and cultural divide (disagreements over public health policy). Although 78% identified at least one positive, less than half (42%) did so at three or more time points. Positive experiences were more diverse across time, but some reported greater interpersonal connection by utilizing technology to increase social contacts. LTA revealed three stances towards positive experiences: active efforts, appreciative efforts (observation), and mixed efforts. While trait resilience was unrelated to the themes, the mixed approach towards positive experiences was associated with lower anxiety at the last assessment, emphasizing the importance of positive experiences during stress.

## 1. Introduction

Despite their greater physical vulnerability to the COVID-19 virus, older adults’ mental health showed surprising resilience. While they did experience a slight increase in psychological symptoms, it was much less than that of younger adults (American Psychological Association, 2021). Reasons for this resilience are at this point largely unknown, but there are suggestions of the use of more adaptive coping strategies than younger adults (Fuller & Huseth-Zosel, 2021). We hypothesize that the well-known positivity bias among older adults may also account for this greater resilience (Carstensen et al., 2011), given the importance of positive affect for the maintenance of well-being under stress (Tugade et al., 2021; Zautra, 2003). We previously used qualitative data to understand the types of difficulties and positive experiences that old adults experienced during the initial weeks of the COVID-19 pandemic (Igarashi et al., 2022). We expanded that study to examine trends in difficulties and positive experiences across eight weeks using longitudinal thematic analysis (Audulv et al., 2022). We also related those themes to baseline resilience, as well as anxiety and depressive symptoms at baseline and the last week of data collection.

### 1.1. Resilience, Positive Affect, and Aging

It is well established that older adults increasingly focus on positive affect and experiences to regulate their emotions (Carstensen et al., 2011), with certain caveats. For example, Almeida et al. (2020) have argued that this may be more characteristic in the current cohort of older adults, but that negative trends in well-being in the current mid-life cohorts call into question whether this will be characteristic of future generations of older adults. In addition, this emphasis on positive emotions may be less true among the old-old (Baltes & Smith, 2003; Aldwin et al., 2014). Nonetheless, it is a reasonable hypothesis that the ability of the current older cohorts to focus on the positive may have contributed to their apparent greater well-being during the pandemic. Indeed, Ford et al. (2021) found that older adults were more likely than younger adults to identify positive aspects of the initial phase of the pandemic.

Positive affect (PA) is an important component of resilience (Troy et al., 2023; Tugade et al., 2021). Zautra (2003) argued that resilience can be seen as the ability to maintain positive affect in the face of stress (see also Folkman et al., 1997). Being able to recognize and/or seek positive experiences during stressful situations may be one way of maintaining PA. Further, positive emotions can promote resilience under stress by promoting better emotion regulation (Tugade & Fredrickson, 2004), as well as self-efficacy (Pillay et al., 2022). It can also enhance social connectedness (Li et al., 2024).

### 1.2. Resilience and Positive Affect During the COVID-19 Pandemic

The COVID-19 pandemic was particularly challenging because it involved a largely uncontrollable stressor that could have traumatic consequences in terms of serious illness, disability, and mortality. This was particularly true at the beginning of the pandemic, when death rates were extremely high in late life (Tejada-Vera & Kramarow, 2022), given the lack of adequate treatment and effective vaccines. Attempts to slow the rate of transmission by requiring the closure of non-essential businesses and self-isolation in homes led many individuals to worry about the adverse health effects of isolation in older adults (Taylor et al., 2020).

Thus, some have suggested that positive affect played an even greater role during the COVID-19 pandemic (Kaye-Kauderer et al., 2021; Verdolini et al., 2021), especially for older adults (Lapierre et al., 2023). For example, Witzel et al. (2025) found that positive experiences were protective against cognitive decline among the old-old during the pandemic. Nonetheless, relatively few studies have examined the role of positive experiences in resilience for older adults during COVID-19 (Igarashi et al., 2022) or how positive experiences related to resilience and well-being. Two studies found patterns of affect, distress, and well-being that lead them to conclude that older adults were more resilient, but neither measured resilience (Li et al., 2024; Schäfer et al. (2022). While Kurth et al. (2024) did examine trait resilience and demonstrated that it affected hassles trajectories in older adults during the pandemic, they did not assess positive affect. Two other cross-sectional studies did have measures of resilience and positive affect, and found both mediated and moderated effects, but these samples were not specific to older adults (Ke et al., 2022; Zhang et al. (2021). Thus, there is a dearth of quantitative studies examining the relationship among resilience and positive affect in older adults during the COVID pandemic.

### 1.3. Qualitative Longitudinal Studies of Older Adults During the COVID-19 Pandemic

There have been a few longitudinal, qualitative studies of older adults during the pandemic, but they have tended to have very small samples. For example, Bloom et al. (2022) identified 35 older adults (80+), part of an ongoing study of aging, who had been interviewed by telephone two to three times during the second year of the pandemic, but data from only 12 participants were utilized. Five themes of problems faced were identified: (1) shopping and food; (2) activities limitations; (3) healthcare disruption; (4) social and psychological problems; and (5) coping. Similarly, Maison et al. (2021) also interviewed 20 individuals (aged 25–55) over the course of the first year of the pandemic and also identified five problem themes: (1) limited contact with others; (2) movement restrictions; (3) changes in active lifestyle; (4) boredom and monotony; and (5) uncertainty about the future. Neither of these studies examined resilience, positive affect, or experiences.

Brooks et al. (2022) conducted four interviews with 12 older adults (aged 65+), and found three themes. The first was related to struggling with loss of social connections, freedoms, and activities. However, the second involved adaptation and how they tried to maintain well-being through participation and connection. The third was appreciation: “enjoying what they had, and finding pleasure and contentment.” Similarly, Boatswain-Kyte et al. (2025) conducted a small study of 10 older Black adults using focus groups. They found considerable evidence for adversity and mental health challenges, including loss, grief, barriers to accessing services, and intergenerational tensions. However, they also found “remarkable resilience,” as individuals drew upon their faith and community networks.

Finally, Ooi et al. (2023) interviewed 40 younger (aged 13–24) and 28 older adults (70+) in the United Kingdom during the first year of the pandemic, focusing on coping and positive experiences. Using qualitative thematic analysis, they identified six themes. The first three reflected engagement in self-fulfilling activities, personal growth, and an increased sense of social cohesion. The remaining themes focused more on the use of targeted problem-focused strategies, emotion regulation strategies, and the giving and receiving of social support.

### 1.4. Present Study

While older adults were seemingly more psychologically resilient than younger adults during COVID-19, there are few empirical studies pinpointing the sources of that resilience. The quantitative studies in older adults reviewed above demonstrated resilience-type patterns in affect and well-being, but did not actually assess trait resilience. The few qualitative studies focused on identifying problems for older adults during COVID, although three did find positive experiences, which again suggested resilience, albeit indirectly. Further, they generally utilized very small samples, limiting generalizability, and did not conduct mixed-method analyses to directly examine whether these experiences either reflected or contributed to participant perceptions of their own resilience.

Thus, the purpose of this study was to examine the extent to which older adults could identify both difficulties and positive experiences during the COVID-19 pandemic, as well as to identify longitudinal themes that may emerge, given changes in how the pandemic was managed, both in terms of social requirements (e.g., social isolation) and individual adaptation. We also examined the relationships between demographics, positive experiences, trait resilience, and well-being. Given that qualitative studies are generally exploratory in nature, we focused on four research questions. Our emphasis was on the positive experiences, given the paucity of research in this area, but we used the difficulties data as a comparison tool.

Research Question 1 addressed COVID-related difficulties and positive experiences.

(a) To what extent were older adults able to identify difficulties and positive experiences related to COVID-19?
(b) What were the types of difficulties and positive experiences reported?

Research Question 2 addressed longitudinal themes for difficulties and positive experiences.

(a) Did longitudinal themes emerge for both difficulties and positive experiences?
(b) Did these patterns show consistency or diversity in the types of difficulties and positive experiences?
(c) If consistencies occurred, did any of these themes change in their intensity across time, i.e., did problems become more severe or abate?

Research Question 3 explored whether demographics (e.g., age, gender, education) were related to the longitudinal themes found for difficulties and positive experiences.

Research Question 4 explored the relationship between the longitudinal themes, trait resilience, and psychological well-being. We examined whether preexisting personal and mental health characteristics, e.g., trait resilience and psychological symptoms at baseline, correlated with the difficulties and positive experience themes. We also examined the relationship of the themes to mental health at the end of the study period.

## 2. Methods and Materials

### 2.1. Procedure

Participants were recruited from the LIFE (Linking Individuals, Families, and Environments) Registry, a subject pool of individuals 50 years and older who reside in Oregon, which is managed by the Center for Healthy Aging Research at Oregon State University. We sent a link for the online Qualtrics surveys, concerning COVID-19 experiences, to individuals who had valid email addresses on file (N = 640). Our research was in line with the Declaration of Helsinki (World Medical Association, 2025). Informed consent information constituted the first page of the questionnaire, and respondents were allowed access to the questions only if they pressed a button indicating consent. The study was approved on 27 April 2020 by the Oregon State University Institutional Review Board (IRB-2020-0637).

Baseline data were collected between 28 April and 4 May 2020; the response rate was 39% (*n* = 247). Respondents who completed the baseline survey were more educated than those who did not return the survey (*X*^2^ = 25.04, *p* < 0.001), but there were no age, gender, or race/ethnicity differences compared to non-responders. As part of the baseline survey, participants were asked if they were interested in participating in further surveys. We sent links to these participants for the next seven weeks. Participation in these surveys was high: on average, respondents completed six of the possible eight surveys (*M* = 5.8, *SD* = 2.7).

### 2.2. Analytic Samples

**Figure 1:**
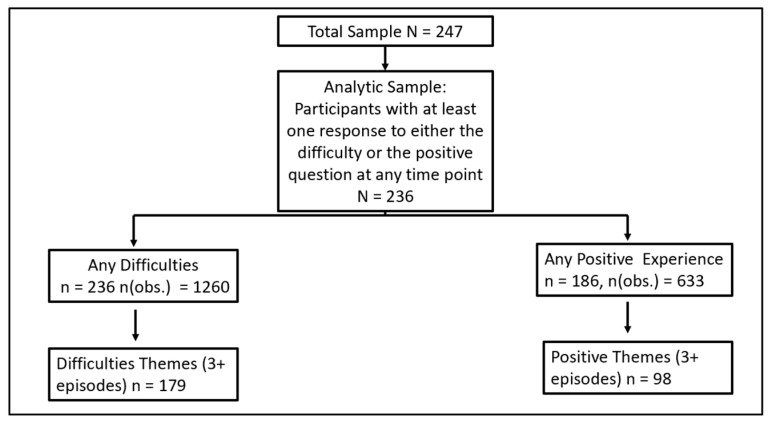
Flow Chart Illustrating Subsamples.

The analytic samples for this study were drawn from the 236 individuals who provided a response to any of the open-ended survey items used in this analysis at any point across the entire study period. Due to the nature of the qualitative data, different subsamples were analyzed for the research questions. Figure 1 illustrates the process of creating the analytic samples.

For RQ1, what types of difficulties and positive experiences were reported and how their frequencies changed over time, the entire analytic sample was used. Women comprised 74% of the sample. Ages ranged from 51 to 95 years (*M* = 71.1, *SD* = 7.3). The majority of participants identified as White (88.7%), which is similar to the United States Census Bureau’s (2020) findings for Oregon (86.7%). Most respondents were married (74%), retired (79%), and well educated: 54% had at least a bachelor’s degree. Given that this constituted 95% of the overall sample, it is not surprising that the analytic sample did not differ from the whole sample by age or any demographic characteristics.

Second, the whole analytic sample reported at least one difficulty at any point during the study period (n = 236; n_obs_ = 1260, M = 5.34, SD = 2.71, range = 1–8). In contrast, only 186 individuals reported at least one positive experience (n_obs_ = 633, M = 3.40, SD = 2.29, range = 1–8). This subsample did not differ significantly on age, sex, race/ethnicity, education, or trait resilience from those (n = 50) who never reported a positive response.

For analyses related to longitudinal themes (RQs 2–4), samples were restricted to participants who reported either difficulties or positive experiences at three or more time points. This resulted in 179 respondents for difficulties and 98 for positive experiences. These subsamples did not differ significantly from the excluded individuals (those with 0–2 episodes in each category) on demographics or trait resilience..

### 2.3. Measures

**Difficulties and Positive Experiences.** Two open-ended questions were asked as part of every weekly survey. The first question concerned difficulties: *During the past week, what was the most difficult thing for you about the COVID-19 situation?* Note that we used the term “difficult” because experience has shown that older adults are less likely to respond to terms like “problems” or “stress,” but will admit to difficulties (see Aldwin et al., 1996), Thus, we used this terminology throughout this manuscript. The second concerned positive experiences: *During the past week, did anything positive come about because of the COVID-19 situation? If yes, explain.* These data underwent substantive content coding as well as thematic analysis (see Analysis section). We also created two variables, difficulties and positive experiences, to indicate whether or not a respondent had ever reported these types of episodes at any point in the eight-week study period.

**Trait Resilience.** The Brief Resilience Scale (BRS; Smith et al., 2008) is composed of six items concerning the individual’s perceived ability to respond to adversity. The scaling of each item ranged from strongly disagree (1) to strongly agree (5). Three items were reverse-scored. Items were summed, with higher scores reflecting a greater sense of resilience: M = 23.31, SD = 4.10, range: 9–30, ∝ = 0.88. This measure was assessed only at baseline.

**Psychological Well-Being.** We assessed psychological well-being throughout the study. For purposes of this analysis, we focused on baseline symptoms to determine if these were reflected in any of the longitudinal themes. We also included well-being measures at the last observation to determine if the longitudinal themes might be influencing well-being, with the due caveat that correlations cannot determine causation.

**Anxiety Symptoms** during the past week were assessed by the Patient-Reported Outcomes Medical Information System (PROMIS) Scale v. 6a (Pilkonis et al., 2011). All six items were rated on a five-point scale that ranged from 0 (never) to 4 (always) and were summed to produce total scores, with higher scores indicating higher levels of anxiety symptoms (baseline: *M* = 2.8, *SD* = 3.3, range = 0–17, α = 0.86; last observation: *M* = 2.32, *SD* = 3.21).

**Depressive Symptoms** during the past week were measured by the PROMIS Scale 6a (Pilkonis et al., 2011). Each of the six items was rated on a five-point scale, ranging from 0 (never) to 4 (always). Overall scores were obtained by summing across ratings given to all items, where higher scores indicate more depressive symptoms (baseline: *M* = 2.4, *SD* = 3.0, range = 0–16, α = 0.84; last observation: *M* = 2.17, *SD* = 2.96).

**Demographics.** All demographic variables were assessed only at the baseline survey. **Age** was calculated by subtracting assessment year (2020) from participants’ birth year. Gender **(female)** was coded as 1 = male and 2 = female. **Educational attainment** was highly skewed and was coded into as 1 ≤ bachelor’s degree and 2 = advanced degrees (master’s or doctorate). **Race/ethnicity** was also dichotomized into non-Hispanic white and people of color (e.g., non-Hispanic Black, Hispanic white).

### 2.4. Mixed-Methods Analyses

The present study utilized a convergent mixed-method design, as qualitative and quantitative data were collected simultaneously, but analyzed separately before being combined in the final analyses (Creswell & Plano Clark, 2018). Qualitative analyses were used to examine the types (RQ1) and longitudinal change in difficulties and positive experiences (RQ2). Integration (i.e., analyses that combined the qualitative and quantitative data) occurred for analyses of the final two research questions to examine demographic (RQ3) and psychological well-being (RQ4) differences in the longitudinal themes for difficulties and positive COVID-19 experiences. The specific analytic approaches are described below.

### 2.5. Qualitative Analyses

Qualitative analyses occurred in two stages, which were completed by three coders. The two primary coders were a non-Hispanic white female in her late 60s and an Asian American female in her mid-60s. The third coder, a non-Hispanic white female in her late-20s, provided input. All three have extensive experience with qualitative methods in midlife and older adult samples.

First, we examined the weekly responses for both the difficulties and positive experiences through directed content analysis (Hsieh & Shannon, 2005). All coders engaged in open coding to gain an understanding of the type of responses provided, guided by earlier work on the baseline data (Igarashi et al., 2022). When responses did not fit within the baseline coding schemes, new codes were proposed and ultimately incorporated into the consolidated scheme used for independent coding (Supplementary Tables S1 and S2).

The second stage of qualitative analyses concerned identifying the longitudinal themes within difficulty and positive experience responses. The thematic analyses (see Audulv et al., 2022) only included 179 respondents who reported difficulties at least three time points and the 98 respondents who reported positive experiences at least three times. Coders 1 and 2 completed independent coding for both stages using ATLAS.ti v.26 (ATLAS.ti Scientific Software Development GmbH, 2026). Multiple codes were often necessary to capture the entirety of these older adults’ experiences.

We then used thematic analysis to code for whether individuals were consistent or diverse in their experiences. Consistent themes were assigned if individuals reported the same difficulty in over half of their content codes. Note that respondents could have more than one consistent theme. Whether or not a difficulty was increasing was judged by the intensity of the language used, such as the underlying tone, the use of all capitals, and punctuation such as exclamation points (especially multiple ones). If the responses for a particular person did not meet the consistency criteria because of the variety of their statements, then the individual was assigned the *Diverse* theme. We used Krippendorff’s (2019) cu-α to determine agreement rates. Disagreements were discussed to resolution among all three coders.

In qualitative analysis, it is important to establish the credibility, transferability, confirmability, and dependability of the analysis (see Elo et al., 2014; Nowell et al., 2017). Credibility was addressed through using multiple independent coders, as well as the congruency between participant responses with reflexive coding (e.g., tracking personal values; Birks et al., 2008). This process was documented by coder 2, who maintained analytic memos documenting the coding and resolution process. Second, transferability (how well our analyses generalize; Elo et al., 2014) was addressed by providing a detailed sample description, as well as the timeframe in which responses were collected, especially important in an ongoing crisis like the pandemic. Third, confirmability was addressed using direct quotations, thereby showing the range of responses within each code. Finally, we have provided a detailed description of the coding to illustrate the dependability of our research (see Supplementary Tables S1 and S2).

### 2.6. Quantitative Analysis (Integration)

We transformed the qualitative codes into dichotomous, quantitative variables for the mixed-method analyses which were conducted in SAS v. 9.4 (SAS Institute Inc., 2014; https://documentation.sas.com/doc/en/pgmsascdc/9.4_3.4/statug/titlepage.htm). For this study, we focused only on the thematic analyses in the mixed-method analyses, as we were interested in how response types/patterns across time were related with psychological well-being. Given that the qualitative analyses were largely exploratory, we opted for bivariate quantitative analyses to conduct a preliminary mixed-method examination of these relationships.

To examine these associations, we used a series of point-biserial correlational analyses. Pairwise deletion was used to account for differences in difficulties and positive experiences response sample sizes. As a reminder, we utilized data from both the baseline and the respondents’ last assessment. Baseline demographics, trait resilience, and depressive and anxiety symptom scores were used to examine whether the difficulties and positive experiences themes that emerged could reflect these baseline characteristics. We also included depressive and anxiety symptom scores from the last assessment in the correlation matrix to examine whether the themes were related to mental health at the end of the study period.

## 3. Results

### 3.1. Research Question 1: Frequency and Types of Difficulties and Positive Experiences

**Figure 2:**
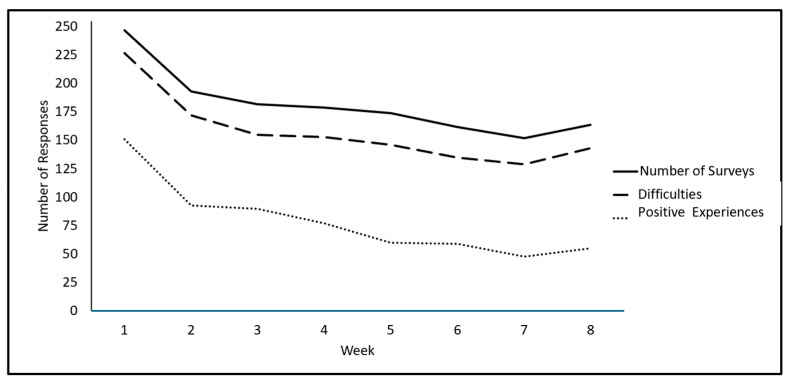
Change in the number of COVID-related difficulties and positive experiences across time.

As Figure 2 shows, reporting of difficulties was quite high, and 79% reported at least one positive experience. The reporting of both difficulties and positive experiences decreased over time, but the decrease appeared slightly steeper for positive experiences. However, there was a slight increase in difficulties from week 7 to 8, partially reflecting social justice concerns stemming from the murder of George Floyd and subsequent social unrest (see Eichstaedt et al., 2021).

**Directed Content Analysis for Difficulties.** Nearly all (95%) of the 247 respondents provided at least one COVID-related difficulty, with 1260 difficulties reported across the eight weeks. There were 14 codes in the final coding scheme, which were distributed over three ecological levels: personal, interpersonal, and society (see Igarashi et al., 2022). Agreement between coders was moderate (Krippendorff’s cu-α = 0.714). Supplementary Table S1 presents the codes and the coding criteria, as well as the number of times the code was applied. The difficulties that were most frequently reported included *Difficulties with EPA* [everyday protective activities] *and Consequences, Psychological Distress, Interpersonal Connections* and *Cultural Divide*.

Five new codes emerged in the longitudinal analyses (see Supplementary Table S1). Some of these codes reflected the considerable confusion of the first few weeks of the pandemic, including problems with *Information* *Assessment*, given the often conflicting and rapidly changing information at the beginning of the pandemic. *Managing Risk* *Assessment* emerged a few weeks later as lockdown was beginning to lift and individuals were trying to balance the pleasure of social interaction with the risk of infection with COVID-19. *Adjustment* also emerged, as individuals developed routines to manage the exigencies of the pandemic. *Interpersonal Conflict* appeared towards the middle of the study as individuals developed “cabin fever” from remaining in close quarters. *Social Justice* concerns emerged, with many respondents commenting on the death of George Floyd, which occurred during the fifth week of the project, as well as the ensuing social protests (see Eichstaedt et al., 2021).

**Directed Content Analysis for Positive Experiences**. The majority of respondents (79%) identified at least one positive experience across the eight weeks, with a total of 633 responses. We identified eight novel codes that were not in the baseline coding scheme (Igarashi et al., 2022). Prior to independent coding, similar codes were consolidated, for a final total of 14 codes (see Supplementary Table S2 for codes, criteria, and frequencies). These codes were also distributed across ecological levels, and agreement was acceptable (Krippendorff’s cu-α = 0.68). The most prevalent positive events concerned *Appreciating Family and Friends, Enjoying a Slower Pace*, experiencing a *Sense of Community* and *Keeping Busy* (with projects around the home and in the community).

The new personal-level codes included simply being *Happy to Stay Home*, which involved not having to commute, enjoying one’s home, and spending time with one’s spouse and pets. Others took to *Lifelong Learning* of new skills and knowledge. *Increasing Self-Confidence* reflected individuals’ newfound ability to cope with the exigencies of the pandemic. Finally, *Tempered Feelings* emerged, as individuals found some experiences to be bittersweet, for example, finally getting to see close relatives, but realizing that they could not hug them for fear of transmitting the virus.

The new interpersonal-level codes included being *Happy for Family and Friends* (often that they had escaped or survived COVID) and *Receiving/Providing Social Support*, with more individuals saying how they had helped others in family and communities. At the societal level, many were overjoyed at the *Lifting of Restrictions* toward the end of the eight-week period, and were able to attend church, go to the gym, get their hair cut, and see family and friends. This presented new opportunities, but also challenges. For example, individuals weighed the benefits of travel to see friends and relatives with greater increased risk of contracting the COVID-19 virus.

### 3.2. Research Question 2: Thematic Analysis of Difficulties and Positive Experiences Across Time

For both sets of analyses, we restricted the samples to those who reported three or more episodes reporting on difficulties and positive experiences, respectively.

**Thematic Analysis of Difficulties.** We identified seven different themes for COVID-related difficulties (see Table 1 for codes, frequencies, and quotations), and agreement was acceptable (Krippendorff’s cu-α = 0.71). Themes were also divided into three ecological levels (although the *Diverse* category could span levels). While most respondents were consistent in both the type and intensity/severity of their difficulties across time, others showed clear longitudinal change, typically with increasing severity/intensity.

At the personal level, about a quarter of the respondents consistently reported *Problems with EPA*, which was the most prevalent content code across all of the difficulty responses. This often extended to more than just discomfort with masks. As one respondent remarked, “*Having to pay attention to safety PPE [personal protective equipment] and practices whenever I go out as if each item or person was a possible hazard*” (65-year-old male, ID 122). About 15% showed consistent psychological distress, while an additional 8% showed evidence of increasing distress. For example, one person’s main difficulty at Time 1 was disrupted physical activity, but by Time 6, they said, “*There is no end in sight. There is no treatment. There is no vaccine. We have no idea how long this major disruption will last*” (70-year-old female, ID 74). While only very few showed consistent adjustment, about 10% showed increasing adjustment. For example, one individual’s main difficulty at Time 1 was being cooped up, but at Time 6 stated, “*This is all getting a bit old, but realized it is necessary*” (89-year-old female, ID 90).

At the interpersonal level, the most frequent theme was *Consistent Problems with Social Connection*, usually missing family and friends. Others were not greatly affected at the start of the study, but developed greater feelings of isolation as the weeks wore on. For example, one woman reported only “*Social distancing*” at Time 1, but by Time 8 stated “*Isolation and not being able to be totally responsible for my own needs*” (80-year-old female, ID 32).

At the societal level, 16% of the sample were consistently experiencing cultural divide, including a 74-year-old female who shared: “*To see the devastation to families who have lost their livelihoods and businesses that are going under every day that this shutdown continues. Too much losing of our civil rights…censoring of conservative viewpoints in the liberal media.*” Others had *Consistent Community Concerns*, who were worried about the COVID-19 mortality rates, the toll it was taking on first responders, and overall concern about their immediate environment. As one 58-year-old female (ID 76) commented, “*Still not being able to attend worship services. It’s still a strange vibe when shopping for groceries…. signs everywhere telling us where to stand, what to wear, don’t sit here, don’t idle here, keep moving, don’t touch that, don’t touch this, ugh! I feel badly for the kids graduating. I feel badly for the families who have lost loved ones.*”

The remaining 14% of the sample could not be characterized by consistency, but instead reported *Diverse* problems across time. For example, at Time 1, one woman complained about “*Lack of spontaneity*,” while at Time 8, they said, “*Balancing desire to begin to reconnect and facing reality of increased COVID in the community and state*” (67-year-old female, ID 130).

**Thematic Analysis of Positive Experiences.** We used an emergent process to identify themes for positive experiences using open coding. As we were engaged in coding, patterns of approaches to positive experiences first emerged. We created three “process” themes that reflected the respondents’ stance towards positive events. In addition, we also created eight “content-focused” themes that were more closely related to the positive experiences described in the l14 longitudinal content codes (see Supplementary Table S2). These themes spanned two ecological levels, personal and interpersonal, although the diverse and other codes could include larger societal concerns. Independent coding consisted of applying all 11 themes (see Table 2), and agreement was acceptable (Krippendorff’s cu-α = 0.71).

The three process themes provided interesting insights into the approaches the respondents took towards positive experiences during the pandemic. A little over half of the sample actively sought positive experiences (*Active Efforts to Pursue the Positive*). For example, one respondent remarked, “*I got all my credit cards paid off. I have refinanced my home, I hope. I have gotten a huge amount of yard work done*” (69-year-old female, ID 43).

A fifth of the respondents were content to find the positive by observing others’ behaviors and practices (*Appreciative Observations*), often remarking on the altruistic behavior of others. As one 73-year-old female (ID 190) shared, “*Not for me personally, but hearing about so many generous gestures some people have performed for others IS positive. Come to think of it, I continue to be humbled by the willingness of two friends to shop for us. Where would we be w/o them??*” The remaining quarter reported a mixture of both active effects and appreciative observations (*Mixed Active Efforts and Observations*): “*I connected with several good friends and shared our thoughts; I talked with family members who need encouragement, I read some great accounts of people who are doing so much to help others and was lifted up by these stories and the efforts so many are doing to save lives and help families, workers and businesses survive*” (77-year-old female, ID 40).

Four of the content-related themes reflected positive experiences at the personal level. Respondents reported enjoying the *Gift of Time*, while others were *Keeping Active and Engaged*. “*Because I’m staying home, household projects are being completed. I learned the digital workflow app, Slack. I volunteered for Mask-match to coordinate donations of personal protective equipment for healthcare workers*” (61-year-old female, ID 37). A few individuals reported *Self Care* activities, which included “*Continued increased physical activity. Attending zoom classes for spiritual, emotional, and physical health. Taking piano weekly lessons over phone, family and friend zoom sessions, having nephew over for social distancing dinner*” (68-year-old female, ID 48). A similar number reported *Self-Enhancement*, as evidenced by a 73-year-old (ID 26) who shared that she had “*More self-awareness and reflection; more time for longer conversations with distant family/friends. More time to do whatever I want to do without the constraints/demands of others.*”

At the interpersonal level, about a fifth of the respondents reported *Greater Interpersonal Connection* (22%), despite the lockdown. A 74-year-old female (ID 154) reported that:

*All of our children live far away from us and far away from each other. They are good about phoning us once a week or so, but we only see them once a year. During this time, via a group text, we are talking with one another off and on throughout the day. They send pictures and jokes. They talk about their activities and work. It has been great to "hear" them talking to each other and great to be in closer touch.*

Another fifth of the respondents found positive experiences within their communities, which we termed *Communitas* (feelings of togetherness in a community setting): “*I have received nice gestures from the people in our community and have heard and read of many positive things that others are doing to help each other. It is heart-warming to see so many people help in so many large and small ways. In spite of all of the difficulties, much has happened to renew faith in humanity*” (77-year-old female, ID 40).

Some respondents identified varying experiences across weeks, and the most frequent of these longitudinal themes was *Diverse*. For example, at Time 1, a 73-year-old male (ID 108) remarked, *Curtailing expenses has helped our bottom line,* and at Time 4, he mentioned, “*Getting connected with my siblings who do not live close by was a positive thing indeed*” (D101).

### 3.3. Research Question 3: Associations with Demographics

The qualitative themes for both difficulties and positive experiences were transformed into dichotomous, quantitative predictors for the final two sets of analyses. Older adults were slightly more likely to answer the qualitative questions (*r* = 0.13, *p* < 0.05), but age was not associated with any of the longitudinal themes for either difficulties or resilience. However, there were some marginal trends with the positive experience themes. For example, age was inversely associated with *Active Efforts* but positively associated with *Mixed Active and Appreciate Efforts*, (*r*s = −0.20 and 0.20, *p*s = 0.06), respectively, as well as *Diverse* (*r* = 0.14, *p* = 0.07). There was a slight negative correlation with anxiety symptoms at the last report (*r* = −0.14, *p* < 0.05).

Women were slightly younger than men (*r* = −0.21, *p* < 0.01) and had slightly higher depressive symptoms at both baseline and the last report (*r*s = 0.15, *p*s < 0.05), as well as higher anxiety levels (*r* = 0.22 and 0.21, *p* < 0.01, respectively). Women were also slightly more likely than men to face *Increased Isolation* and *Increased Psychological Distress*, (*r*s = 0.16, *p* < 0.05), and they reported slightly lower levels of resilience (*r* = 0.12, *p* = 0.05).

Race/ethnicity was also correlated with higher anxiety levels at both baseline and last report (*r =* 0.18, *p* < 0.01, and 0.15, *p* < 0.05, respectively). It was also more likely to be associated with *Increasing Isolation* (*r =* 0.28, *p* < 0.01) and inversely associated with *Diverse* themes for both difficulties and positive experiences (*r*s = −0.16, *p* < 0.05).

Education was associated with fewer depressive symptoms at baseline (*r* = −0.16, *p* < 0.05), as well as *Consistent Problems with Social Connections* (*r* = 0.19, *p* = 0.01). Finally, trait resilience at baseline was moderately related to both depressive and anxiety symptoms at baseline and the last report (*r*s ranged from −0.33 to −0.44, *p* ≤ 0.01).

### 3.4. Research Question 4: Are Resilience and Psychological Well-Being Associated with the Difficulties and Positive Experiences Themes?

Table 3 presents the correlations between the difficulties and positive experiences themes and baseline resilience. Psychological well-being at baseline and the last reporting was assessed using depressive and anxiety symptoms, both at baseline and at the last reporting (i.e., the last survey with qualitative data).

**Difficulties Themes.** As nearly all of the respondents reported at least one problem during the study period, it is not surprising that there was no correlation between resilience or any psychological well-being predictors and the ability to respond to this open-ended item. Further, resilience was not significantly related to any of the difficulties longitudinal themes. There was a small correlation between *Consistent Psychological Distress* and baseline depressive symptoms, *r* = 0.16, *p* < 0.05, while *Increasing Psychological Distress* evidenced modest associations with the final depressive and anxiety symptom levels (*r*s = 0.16 and 0.19, *p* < 0.05, respectively). The only other difficulties theme to evidence an association with psychological well-being was *Diverse*, which was negatively associated with baseline depressive and anxiety symptoms (*r*s = −0.15, *p* < 0.05).

**Positive Experiences Themes.** Similar to the difficulties themes, responding at least once to this open-ended item on positive experiences was not associated with resilience or with any of the psychological well-being variables. This was also true of the content-related positive experiences themes. However, the process themes were associated with well-being at the last reporting. Surprisingly, the *Active Efforts* theme was positively associated with anxiety symptoms at the last report (*r* = 0.21, *p* < 0.05), while the *Mixed Active and Appreciative Efforts* theme was negatively associated with anxiety (*r* = −0.21, *p* < 0.05).

## 4. Discussion

Given the importance of positive affect and experiences in studies of resilience (Ke et al., 2022; Tugade et al., 2021), we sought to understand the types of positive experiences that older adults reported, using qualitative data, over eight weeks at the beginning of the COVID-19 pandemic. During this time, there were no adequate treatments, no vaccines were available, and lockdowns were in place. Only a handful of other studies had examined this issue, and those typically had very small samples. We included reporting of COVID-related difficulties to provide a context.

### 4.1. Qualitative Analyses

Some of the difficulties we identified were similar to those found by other qualitative studies (Bloom et al., 2022; Maison et al., 2021), such as problems with EPA, psychological distress, and struggles with interpersonal connections. However, we had a broader range of problems, undoubtedly due to our larger sample and the fact that we had a longitudinal design that allowed us to track emerging and novel problems.

For example, a novel difficulty was the cultural divide, which reflected the political divisiveness over public health policies. Both sides of the political divide were often unhappy with how governments were handling the crisis, either due to economic damage from the lockdown or from a lack of clear guidance for public health measures. They also disagreed about masking and social distancing requirements, with some lamenting perceived difficulties in the community due to unnecessary restrictions and others upset at the lack of adherence to public health mandates, putting them at risk. This has received little attention in previous qualitative (or quantitative) studies of stress in older adults during the pandemic, although some work has been done among young adults (e.g., Howard et al., 2023).

Other novel difficulties we identified included interpersonal conflicts emerged as the pandemic wore on, as did problems in information assessment and the management of long-term risk. With gradual lifting of the lockdown, some debated the wisdom of visiting loved ones for fear of getting COVID-19 or conversely giving it to frail parents or young grandchildren. Finally, with the death of George Floyd, social justice issues arose, with some stating their dismay at the police brutality and others upset that social protests were allowed, but not other group activities such as church attendance (see Eichstaedt et al., 2021). Scotland et al. (2024) found a similar divide in Facebook posts on a Black Lives Matter page.

In contrast, the positive experiences, although reported by fewer people, were more diverse. By far the most frequent positive experience was appreciating family and friends, which was also found by Ooi et al. (2023). The respondents in this sample went to great lengths to maintain contact with others. They developed new skills, like Zooming with friends and family and participating in group texts with their adult children.

However, most of the positive experiences that emerged from this study were novel and not reported by previous studies. Particularly noteworthy was the importance of communitas. Many of our respondents lived in smaller communities, and they often expressed pride and gratitude for how well the communities banded together. Many spoke of participating in the “Mask Brigade,” which was an informal association of citizens who made and delivered masks for healthcare workers, first responders, and others who needed to work during the mask shortage at the beginning of the pandemic (see Adams, 2020). Some also found witnessing other acts of kindness in the community to be inspirational. In contrast, some of the respondents who were most upset at government restrictions thought that the community had changed for the worse, interpreting mask wearing and social distancing almost as a form of shunning or avoidance. They mourned the loss of their communities, largely faith-based. This is also in contrast to Boatswain-Kyte et al. (2025), who found that a major component of resilience in their sample was based on faith-related activities and for whom community was seen more as a source of support.

It was in the process of identifying longitudinal themes that the biggest differences emerged between the difficulties and the positive experiences. The themes identified for difficulties were often consistent across time or showed temporal dynamics, usually increases in things like psychological distress or social isolation. In contrast, we were unable to identify clear temporal dynamics in the positives. While some were consistent in their experiences, others were often quite diverse, but in no circumstances did we see the intensity of the experiences increase, as we saw in the difficulty themes. Rather, we found three process approaches to positive experiences. Some exhibited active approaches, seeking out or creating positive experiences, while others were more likely to observe and be grateful for the acts of kindness they saw around them. Some utilized both approaches. This combination of positive appreciation and action-oriented behavior resonates with the work of Macy and Young Brown (2025). As clinicians helping people address feelings of anxiety and hopelessness over the human-driven destruction of the planet, they identified gratitude as a first step before moving forward with action to address despair.

### 4.2. Mixed-Method Analyses

Age was not associated with social isolation themes, meaning that the older adults in this sample were not more likely to be socially isolated. It was a community-residing sample for the most part, and 70% of the sample was married. This is higher than the <60% rate found in national samples (Loo & Brown, 2024). It should be noted that the older adults in our sample were more likely to be male, who are less likely to be widowed than older women. This sample composition is unusual, but likely reflects the fact that this was an internet survey, and these older males may have been more comfortable with computers than their female counterparts (Lee et al., 2019). The women in this study were more likely to experience increasing difficulties with social isolation and psychological distress, and their lower participation levels among the old-old members of the sample may have affected our age findings.

There were modest associations between the themes and mental health. We examined simple correlations between themes with depressive and anxiety symptoms at baseline and then again at the last report to attempt to understand system dynamics. For the problem themes, we found that depressive symptoms at baseline were associated with consistent psychological distress, which supports previous findings that those with existing mental health problems were more likely to be distressed during the pandemic (Wang et al., 2020). However, the themes of increasing psychological distress and social isolation were associated with higher depressive and anxiety at the last report, suggesting that experiencing these types of difficulties was affecting mental health.

In contrast, for the positive experiences, only the process themes were associated with psychological symptoms. Consistently undertaking an active stance towards (or seeking out) positive experiences was associated with higher levels of anxiety at the last report, while taking a mixed observational and active stance was associated with lower levels of anxiety. Perhaps having to actively seek out positive experiences either reflected or exacerbated anxiety, but appreciating and seeking out the positives appeared to result in lower anxiety levels. Similar to Macy and Young Brown (2025), our study suggests that the experience of gratitude or appreciation in combination with action was important in reducing anxiety rather than action alone.

Most puzzling was the fact that the trait measure of resilience at baseline was unrelated to any of the themes, which contradicts the general assumption that positive experiences are associated with resilience (e.g., Tugade et al., 2021). Resilience was significantly, inversely, and moderately associated with depressive and anxiety symptoms at both time points, suggesting it did play a role in adaptation to the COVID-19 pandemic, just not through the pathways we expected.

Nonetheless, it was clear that our respondents were demonstrating the process aspects of resilience (e.g., coping strategies in dealing with the problem at hand). First, we were struck by the extent to which these older adults sought out and maintained social connections, despite the lockdown. Some remarked that they got in touch with old friends that they had not seen in years, while others joined new interest groups online.

Second, many of our respondents used cognitive reappraisal processes to take a positive perspective of the lockdown, viewing it as a welcome lull in often busy schedules involving caregiving for parents and grandchildren, community volunteer activities, and the like. They welcomed the slower pace of life, and appreciated the decrease in traffic and the resulting reemergence of clean air, birds, and other wildlife. Others perceived the lockdown as a chance to finally tackle long-delayed projects. Some took up new endeavors, for example, finding treasure troves of beloved music on the web, trying to learn a new instrument or language, taking new classes on the web to develop cooking skills, or reigniting long-dormant interests. Yet others used this period for self-improvement, focusing on meditation, yoga, exercise, and journaling to develop insight.

### 4.3. Limitations and Strengths

Like most studies of older adults during the pandemic, we utilized internet survey techniques, which undoubtedly biased the sample towards individuals that may have had better access to resources such as computers and the internet (i.e., higher education). It is likely that the difficulties and positive experiences here may not completely generalize to other samples. For example, Boatswain-Kyte et al.’s (2025) qualitative study of older Black adults during COVID found indications of resilience through relying on their communities and faith, but found much more grief and loss than we did. While our subsamples did not differ on demographics, it is possible that there were other distinguishing characteristics not reflected in those types of statistics, such as personality or unidentified life circumstances. Further, using survey results obviates the ability to pose the sort of clarifying questions often so useful in qualitative research involving interviews.

We should note that our inter-rater reliabilities (ranging from 0.68–0.71) would be characterized as modest to moderate (Krippendorff, 2019). However, that author also stated that reliabilities are adversely affected by the number and complexity of the codes, which we feel is applicable to our situation. Further, all discrepancies were discussed to resolution by all coders. Nonetheless, generalization should be viewed with caution. It would be interesting to see if similar positive experiences emerged with other collective stressors, such as natural disasters, or if these were specific to a fairly protected sample of older adults during the COVID-19 pandemic.

Given the exploratory nature of the qualitative analyses, we used bivariate quantitative analyses in the mixed-method part of the study. Thus, the questions we asked were relatively simple: Did baseline demographics, trait resilience, and psychological symptoms influence the experience of difficulties and positives during COVID, and/or did these experiences relate to psychological symptoms at the end of the study? Having established what the patterns of positive and difficulties were, future research addressing more complex questions utilizing multivariate analytical techniques would be appropriate.

The lack of relation with the trait measure of resilience was disappointing, and it is possible that other measures of resilience may have been better able to capture its relationship with difficulties and positive experiences in these novel stressful circumstances. The current measure relied mainly on the “usual” ability to “bounce back,” which is perhaps less relevant to novel, ongoing pandemics that are not readily amenable to control. Trait resilience measures (i.e., what one usually does) should be developed that are more sensitive to the broader range of strategies found in qualitative process resilience studies that examine what individuals actually do in stressful situations, such as seeking out the positive or learning how to reframe problems.

## 5. Conclusions

Our larger sample and longitudinal design allowed us to identify novel difficulties and positive experiences not reported in previous studies. In particular, understanding the role that community integration plays in positive adaptation should be more prominent in the resilience literature (see Aldwin & Root, in press). Further, measures of resilience reflect an earlier definition as just a personality characteristic. More current theoretical models are more likely to focus on dynamic processes occurring at multiple levels (see Masten, 2021), and our resilience measures need to be reformulated to reflect these newer models and include positive experiences. Finally, we were struck by the process themes that emerged from the analysis of the positive experiences. We were pleased to see how at least some of our respondents were actively seeking out positive experiences, while others were more attuned to appreciating the aspects of their experiences that could be seen as positive (such as community solidarity). However, incorporating both active and appreciative attitudes appeared to be most protective of mental health. To our knowledge, this is a novel finding, and we would encourage future research to replicate and more deeply investigate this phenomenon.

*My only fear was of dying on a ventilator with no one who loves me nearby. I no longer fear that because the time has allowed me to consider all the good things that are happening… The young people are beautiful examples of making the best of bad times. I hope we can learn from their example.*

(86-year-old female, ID 193)

## Figures and Tables

**Table 1:** Longitudinal themes for COVID-related difficulties (N = 179).

Themes	*n (%)*	Quotes from Two Timepoints for Selected Respondents
**Personal Level**		
1. Consistent EPAs	56 *(27)*	**65-year-old male (ID 122)** *T1: “Having to pay attention to safety PPE and practices whenever I go out as if each item or person was a possible hazard.”* *T7: “Not participating in an annual end of the year choral concert since choir has been cancelled since mid-early March.”*
2. Consistent Psychological Distress	31 *(15)*	**70-year-old female (ID 47)** *T1: “Uncertainty about the future, worry about exposure, anger about the lack of federal government response….”* *T5: “Uncertainty about when things will ease up making decision about having hip replacement surgery—worry about encountering the health care system.”*
3. Increasing Psychological Distress	18 *(09)*	**70-year-old female, ID 74** *T1: “The single most difficult problem I have faced with the COVID-19 situation is that my normal exercise routine is completely disrupted.”* *T6: “There is no end in sight. There is no treatment. There is no vaccine. We have no idea how long this major disruption will last.”*
4. Consistent Adjustment	4 *(02)*	**71-year-old male, ID 51** *T1: “Seriously, my life has changed very little due to COVID-19. My biggest “inconvenience” has been that I chose to NOT go to a hardware store for parts as I rebuilt my large greenhouse (damaged by previous snow weight). I had to jury rig a few aspects until I can get to the hardware store. That’s not “difficult” but makes me feel clever and prepared for whatever I need to do—which is a necessary part of normal rural living.”* *T7: “With my life going well and pretty much inside a bubble, I sometimes forget the pandemic is happening at all… There are times when I need to give myself a dope slap to recall the reality of the situation for so many others.”*
5. Increasing Adjustment	21 *(10)*	**89-year-old female, ID 90** *T1: “Feeling cooped up.”* *T6: “This is all getting a bit old, but realized it is necessary.”*
**Interpersonal Level**		
6. Consistent Social Connection Problems	42 (*20)*	**68-year-old female, ID 27** *T1: “How long will this last? How much longer can I make it emotionally with no human touch?”* *T7: “It’s been months since I’ve had a hug or any touch at all.”*
7. Increasing Social Connection Problems	11 *(05)*	**80-year-old female, ID 32** *T1: “Social distancing.”* *T8: “Isolation and not being able to be totally responsible for my own needs.”*
**Societal Level**		
8. Consistent Cultural Divide	33 *(16)*	**74-year-old female, ID 56** *T1: “To see the devastation to families who have lost their livelihoods and businesses that are going under every day that this shutdown continues. Too much losing of our civil rights…censoring of conservative viewpoints in the liberal media.”* *T7 “Let’s open up and let people get back to work!! People are loosing their livelihoods. Too much fear mongering in the media and by so called doctors.”*
9. Consistent Community Problems	5 *(01)*	**58-year-old female, ID 76** *T3: “Still not being able to attend worship services. It’s still a strange vibe when shopping for groceries…. signs everywhere telling us where to stand, what to wear, don’t sit here, don’t idle here, keep moving, don’t touch that, don’t touch this, ugh! I feel badly for the kids graduating. I feel badly for the families who have lost loved ones.”* *T7: “Seeing people out at the stores who swing way wide when you are approaching even if I have a mask on. This social distancing thing has made us all unsocial and afraid! I am so done with it”!*
10. Increasing Community Problems	1 *(00)*	**80-year-old female, ID 134** *T1: “People I know—including a couple relatives—who make insensitive or rude comments about the COVID-19 situation.”* *T8: “Seeing the numbers [of deaths] continue to go up in Oregon and around the country.”*
11. Diverse	28 *(14)*	**67-year-old female, ID 130** *T1: “Lack of spontaneity.”* *T8: “Balancing desire to begin to reconnect and facing reality if increased COVID in the community and state.”*

Note: To demonstrate consistency or change in themes, we presented quotes from two episodes for each theme example, indicating which time they occurred (i.e., T1 = Time 1). Respondents could receive multiple codes, so percentage total may exceed 100%. EPA = everyday protective activity; PPE = personal protective equipment. Krippendorff’s cu-α = 0.71.

**Table 2:** Longitudinal themes for positive experiences (N = 98).

Themes	n *(%)*	Example
**Process Themes**		
1. Active efforts to pursue the positive	52 *(53)*	**69-year-old female, ID 43.** “*I got all my credit cards paid off. I have refinanced my home, I hope. I have gotten a huge amount of yard work done.*”
2. Appreciative observations of the positive	21 *(21)*	**73-year-old female, ID 190.** “*Not for me personally, but hearing about so many generous gestures some people have performed for others IS positive. Come to think of it, I continue to be humbled by the willingness of two friends to shop for us. Where would we be w/o them??*”
3. Mixed active efforts and observations	25 *(26)*	**77-year-old female, ID 40.** “*I connected with several good friends and shared our thoughts; I talked with family members who need encouragement, I read some great accounts of people who are doing so much to help others and was lifted up by these stories and the efforts so many are doing to save lives and help families, workers and businesses survive.*”
**Content-Focused Themes** **Personal**		
4. Gift of time	12 *(12)*	**75-year-old female, ID 37.** “*I wake up and get up earlier, which I’m liking. Also, I love the relaxation of being alone with few responsibilities except to take care of myself.*”
5. Keeping active/engaged	10 *(10)*	**61-year-old female, ID 85.** “*Because I’m staying home, household projects are being completed. I learned the digital workflow app, Slack. I volunteered for Mask-match to coordinate donations of personal protective equipment for healthcare workers.*”
6. Self-care	6 *(6%)*	**68-year-old, ID 48.** “*Continued increased physical activity. Attending zoom classes for spiritual, emotional, and physical health. Taking piano weekly lessons over phone, family and friend zoom sessions, having nephew over for social distancing dinner.*”
7. Self-enhancement	6 *(6%)*	**73-year-old female, ID 26.** “*More self-awareness and reflection; more time for longer conversations with distant family/friends. More time to do whatever I want to do without the constraints/demands of others.*”
**Interpersonal**		
8. Greater interpersonal connection	22 *(22)*	**74-year-old female, ID 154.** “*All of our children live far away from us and far away from each other. They are good about phoning us once a week or so, but we only see them once a year. During this time, via a group text, we are talking with one another off and on throughout the day. They send pictures and jokes. They talk about their activities and work. It has been great to “hear” them talking to each other and great to be in closer touch.*”
9. Communitas	21 *(21)*	**77-year-old female, ID 40.** “*I have received nice gestures from the people in our community and have heard and read of many positive things that others are doing to help each other. It is heart-warming to see so many people help in so many large and small ways. In spite of all of the difficulties, much has happened to renew faith in humanity.*”
10. Diverse	28 *(29)*	**73-year-old male, ID 108.** *T1: “Curtailing expenses has helped our bottom line.”* *T4: “Getting connected with my siblings who do not live close by was a positive thing indeed.”*
11. Other (financial)	3 *(3)*	**73-year-old male, ID 17.** “*Raise in income from unemployment.*”

Note: Respondents could receive multiple codes, so percentage total may exceed 100%. Krippendorff’s cu-α = 0.71.

**Table 3:** Correlations of difficulties and positive experience themes with resilience and mental health.

Theme	Resilience	Depressive Symptoms Week 1	Depressive Symptoms Last Obs.	Anxiety Week 1	Anxiety Last Obs.
Difficulty	0.09	−0.07	−0.06	−0.01	−0.12 ^+^
Consistent EPAs	−0.03	0.08	0.13 ^+^	0.06	0.12
Cons. Psych. Distress	−0.13	0.16 *	0.07	0.12	0.02
Incr. Psych. Distress	−0.05	0.06	0.16 *	0.10	0.19 *
Consistent Adjustment	0.09	0.06	0.13 ^+^	−0.02	0.06
Increased Adjustment	0.03	−0.08	−0.10	−0.01	−0.11
Cons. Social Connection	−0.08	−0.04	−0.01	0.03	0.03
Increased Isolation	0.06	0.00	0.01	0.03	−0.01
Cons. Cultural Divide	0.13	−0.08	−0.12	−0.02	−0.07
Incr. Community Probs.	0.10	0.02	−0.03	0.00	−0.03
Diverse Problems	0.04	0.15 *	−0.08	−0.15 *	−0.05
Positive Experience	0.09	−0.05	−0.06	−0.01	−0.06
Active Efforts	0.09	−0.04	0.16	0.13	0.21 *
Appreciative Efforts	0.05	−0.06	−0.11	−0.12	−0.03
Mixed Active & Appreciative Efforts	−0.15	0.10	−0.08	−0.04	−0.21 *
Gift of Time	−0.05	−0.05	0.03	−0.06	−0.02
Keeping Active	−0.01	0.06	0.19 ^+^	0.12	0.15
Self-Enhancement	0.06	−0.14	−0.08	−0.15	−0.07
Self-Care	0.16	−0.04	0.10	0.00	0.10
Interpersonal	−0.08	0.06	0.13	0.14	0.17 ^+^
Community	0.00	0.00	−0.08	−0.03	−0.04
Diverse	0.07	0.02	−0.12	−0.05	−0.13

NOTE: Pairwise deletion; *n* = 245; difficulty themes *n* = 179; positive experience themes *n* = 98. Numbers may vary slightly due to missing data. Incr. = increasing. Cons. = consistent. EPAs = everyday protective activities. ^+^ *p* < 0.10; * *p* ≤ 0.05; significant correlations are bolded.

## Data Availability

Due to the sensitive nature of the data collected by this study and Institutional Review Board restrictions, only the coding scheme and analytic methods will be made available from carolyn.aldwin@oregonstate.edu.

## Supplementary Materials

The following supporting information can be downloaded at https://www.mdpi.com/article/10.3390/bs16071117/s1. Table S1: Content Codes for Longitudinal Difficulties (N_obs_ = 1260 episodes); Table S2: Consolidated Content Analysis of Positive Experiences (N = 633 episodes).
