# Supplementary material for: Beyond Resilience: A Mixed-Method, Longitudinal Analysis of Difficulties and Positive Experiences in Older Adults During the COVID-19 Pandemic
Source: Behav Sci (Basel). 2026 Jul 3;16(7):1117. doi: 10.3390/bs16071117 (PMC13405987; doi:10.3390/bs16071117)
Supplement: Supplementary file 1 [file behavsci-16-01117-s001.zip › behavsci-4263461-supplementary.pdf]

Supplementary Table S1: *Content Codes for Longitudinal Difficulties (N_{obs} = 1,260 episodes)*

Codes by Ecological Level	*n*	Criteria
**<u>Personal</u>**		
Difficulties with EPAs and Consequences	445	Adjusting to everyday protective activity (EPA), getting EPA supplies, lockdown, social distancing, activity constraint, assessing immediate risk, difficulty accomplishing goals, getting non-EPA supplies, problems with connectivity, professional constraints, major life change in response to COVID-19, weight gain (e.g. forgetting masks or handwashing)
Psychological Distress	374	Future uncertainty, angst, boredom, lack of meaning/ purpose, feeling vulnerable, media burnout (e.g., feeling lack of control), fatigue, strong emotions expressed
Worry About Personal Finances	8	Unemployed, stock market, job security (e.g., continued unemployment)
Managing Long-Term Risk*	72	Concerns re moving on and/or balancing gains vs. risks. Is personal decision often within an interpersonal context. Concerns about opening up. Not immediate risk.
Information Assessment*	36	Not media burnout but just trying to figure out what's going on. Uncertainty about who/what to trust.
Adjustment*	28	Adjustment (e.g., no better but no worse, e.g., getting used to it)
**<u>Interpersonal</u>**		
Struggles with Interpersonal Connections	322	Social isolation, loneliness, human touch (e.g., lack of social contact)
Concern for Close Others	62	Concern for family, fear of hurting/harming others, worrying about adult children (e.g., concern for spouse getting exposed)

Interpersonal Conflict*	44	Arguments with immediate social network, discomfort with their decisions/coping strategies, e.g., too much time together
**<u>Societal</u>**		
Cultural Divide	274	Politics, COVID-19 Everyday Protective Actions, media, challenge to assumption system (e.g., people not wearing masks)
Concern for Society	28	Economic state of society, incompetence of healthcare system, missing opportunity for improving society, vaccine development, worry about society (e.g., when the vaccine would be available)
Reactions to COVID-19 Cases and Deaths	53	Monitoring increasing numbers, anguish over COVID illness/death, death of cultural icons (e.g., increasing number of deaths)
Concern for Community Others	53	Concern for others, having to lay off staff (e.g., concern for other people suffering from COVID-19)
Social Justice Issues*	43	Any mention of George Floyd, Black Lives Matter, riots

* New codes that emerged during longitudinal coding. Krippendorf's cu-α= 714.

Supplementary Table S2: *Consolidated Content Analysis of Positive Experiences (N = 633 episodes)*

Consolidated Codes	N	Criteria
**Personal**<br>1. Keeping Busy	100	Engaging in familiar activities such as cooking, gardening, hobbies, completing projects, cleaning, & organizing home
2. Enjoying a Slower Pace	115	*Freedom of Simplicity*<br>Experiencing fewer constraints & demands from daily life, being relieved of guilt from not doing for others, having more time, living a simpler life, able to slow down, feeling more relaxed<br>*Happy to Stay Home**<br>Experiencing an appreciation for home, spending more time at home, working from home, and having an "excuse" to stay home, include more time with pets
3. Doing Something New	58	Engaging in novel, never-done-before activities including crafts, gardening, cooking, and adopting a dog. Many activities were related to internet-supported activities: dating, music performances, courses, & music group sessions. Include technology, media, etc.
4. Creative Engagement	55	Doing intellectually active things like webinars, lectures, arts & crafts, etc.<br>*Lifelong Learning**<br>Learning **how** to do something
5. Health and Wellness	50	Wellness and fitness-focused activities, such as increased physical fitness, improved nutrition, better sleep, and health maintenance activities
6. Increasing Self-Awareness	37	Engaging in activities to increase self-awareness (meditation, journaling), having time to be reflective, overcoming fears, increasing spirituality

7. Experiencing Gratitude and Appreciation	41	Experiencing gratitude for life situations, awareness of blessings, having everything I need, appreciation of nature. Hopeful on a personal level.
8. Financial Benefits	30	Being employed, receiving economic relief from stimulus check, and improved personal finances from curtailing expenses
9. Increasing Self-Confidence*	23	*Adjusting*<br>Decrease in psychological distress, adapting to new context.<br>*Gaining Experience*<br>Learning from COVID experience<br>*Sense of Purpose*<br>Feeling useful; focus is on individual<br>*Belief in One's Own Resilience*<br>Determination to get through the problem; do what it takes; Know that I can handle things
10. Tempered feelings*	28	Use when positive feelings are tempered by realistic understanding of problems, or for mixed emotions in general, e.g., Need to set aside personal desires for greater good, i.e., not hugging to avoid infecting loved one). Can double code
**Interpersonal**		
1. Appreciating Family & Friends	214	*Valuing Time with Family and Friends*<br>More contact with family and friends (both in-person and remotely via text, internet supported, phone), reconnecting with old friends, greater engagement with others, more time with spouse<br>*Happy for Family & Friends**<br>Restricted to people you know; belief in family's resilience<br>*Receiving/Providing Support from Family & Friends**

		Helping others with specific tasks, e.g., making masks for others; Receiving tangible help with specific tasks, e.g., others go grocery shopping; When others provide information germane to specific problems.
12. Experiencing Community	105	
Sense of Community		Experiencing kindness from others in one's community, wanting to help others, participating in community volunteer efforts such as making masks, witnessing positive community actions, feeling empathy for others experiencing hardship, social solidarity, witnessing improved civility, creating community.
		*Happy for Others in the Community**
		Happy for others in the community, e.g., happy that COVID rates are low, etc.
		*Receiving/Providing Support from non-intimates**
		Receiving/offering offers of help from neighbors, acquaintances, etc.
**Societal**		
13. Macro Optimism	60	
Social Optimism		Feeling hopeful for society, focus on social justice reforms, global solidarity, hopeful for COVID-19 improvements and medical advances
.		
Environmental Optimism		Celebrating cleaner water and air (personal observations and media coverage), feeling the planet is getting a moment to recover, hopefulness for progress in addressing climate change
14. Lifting of Restrictions*	38	Easing of COVID restrictions; anticipating or experiencing local changes

* New content category that emerged during longitudinal coding. Krippendorf's cu-α = .68.
